# Efficient RT-QuIC seeding activity for α-synuclein in olfactory mucosa samples of patients with Parkinson’s disease and multiple system atrophy

**DOI:** 10.1186/s40035-019-0164-x

**Published:** 2019-08-08

**Authors:** Chiara Maria Giulia De Luca, Antonio Emanuele Elia, Sara Maria Portaleone, Federico Angelo Cazzaniga, Martina Rossi, Edoardo Bistaffa, Elena De Cecco, Joanna Narkiewicz, Giulia Salzano, Olga Carletta, Luigi Romito, Grazia Devigili, Paola Soliveri, Pietro Tiraboschi, Giuseppe Legname, Fabrizio Tagliavini, Roberto Eleopra, Giorgio Giaccone, Fabio Moda

**Affiliations:** 1 Fondazione IRCCS Istituto Neurologico Carlo Besta, Unit of Neurology 5 and Neuropathology, Milan, Italy; 2 Fondazione IRCCS Istituto Neurologico Carlo Besta, Unit of Neurology I - Parkinson and Movement Disorders Unit, Milan, Italy; 3 Department of Health Sciences, Università degli Studi di Milano, Otolaryngology Unit, San Paolo Hospital, Milan, Italy; 4 Department of Neuroscience, Scuola Internazionale Superiore di Studi Avanzati (SISSA), Laboratory of Prion Biology, Trieste, Italy; 5 Fondazione IRCCS Istituto Neurologico Carlo Besta, Scientific Directorate, Milan, Italy

**Keywords:** RT-QuIC, Olfactory mucosa, Parkinson’s disease, Neurodegenerative parkinsonisms, α-Synuclein

## Abstract

**Background:**

Parkinson’s disease (PD) is a neurodegenerative disorder whose diagnosis is often challenging because symptoms may overlap with neurodegenerative parkinsonisms. PD is characterized by intraneuronal accumulation of abnormal α-synuclein in brainstem while neurodegenerative parkinsonisms might be associated with accumulation of either α-synuclein, as in the case of Multiple System Atrophy (MSA) or tau, as in the case of Corticobasal Degeneration (CBD) and Progressive Supranuclear Palsy (PSP), in other disease-specific brain regions. Definite diagnosis of all these diseases can be formulated only neuropathologically by detection and localization of α-synuclein or tau aggregates in the brain. Compelling evidence suggests that trace-amount of these proteins can appear in peripheral tissues, including receptor neurons of the olfactory mucosa (OM).

**Methods:**

We have set and standardized the experimental conditions to extend the ultrasensitive Real Time Quaking Induced Conversion (RT-QuIC) assay for OM analysis. In particular, by using human recombinant α-synuclein as substrate of reaction, we have assessed the ability of OM collected from patients with clinical diagnoses of PD and MSA to induce α-synuclein aggregation, and compared their seeding ability to that of OM samples collected from patients with clinical diagnoses of CBD and PSP.

**Results:**

Our results showed that a significant percentage of MSA and PD samples induced α-synuclein aggregation with high efficiency, but also few samples of patients with the clinical diagnosis of CBD and PSP caused the same effect. Notably, the final RT-QuIC aggregates obtained from MSA and PD samples owned peculiar biochemical and morphological features potentially enabling their discrimination.

**Conclusions:**

Our study provide the proof-of-concept that olfactory mucosa samples collected from patients with PD and MSA possess important seeding activities for α-synuclein. Additional studies are required for (i) estimating sensitivity and specificity of the technique and for (ii) evaluating its application for the diagnosis of PD and neurodegenerative parkinsonisms. RT-QuIC analyses of OM and cerebrospinal fluid (CSF) can be combined with the aim of increasing the overall diagnostic accuracy of these diseases, especially in the early stages.

**Electronic supplementary material:**

The online version of this article (10.1186/s40035-019-0164-x) contains supplementary material, which is available to authorized users.

## Background

Parkinson’s disease (PD) is the second most common neurodegenerative disorder after Alzheimer’s disease (AD) and is characterized by bradykinesia with rigidity, tremor and postural instability [1, 2]. Neurodegenerative parkinsonisms resemble PD in some of its clinical feature and include Multiple System Atrophy (MSA), Progressive Supranuclear Palsy (PSP) and Corticobasal Degeneration (CBD) [3, 4]. Although highly heterogeneous from a clinical and neuropathological point of view, all these diseases share a common pathological mechanism that involves the accumulation of abnormally folded proteins in the Central Nervous System (CNS) [5, 6]. In particular, PD is characterized by the presence of α-synuclein aggregates in neuronal perikarya and neuronal process (referred to as Lewy bodies and Lewy neurites) mainly located in the brainstem, while neurodegenerative parkinsonisms can be divided in two molecular classes [7, 8]. The first molecular class corresponds to MSA that is characterized by accumulation of abnormal conformers of α-synuclein (also referred to as α-synucleinopathies) in oligodendrocytes of cerebellum, pons and basal ganglia [3, 9–11]. The second molecular class includes disorders characterized by brain accumulation of abnormal forms of tau protein which cause PSP or CBD (hence referred to as tauopathies). PSP shows aggregates of 4-repeat tau (4R) in neurons (neurofibrillary tangles), astrocytes (tufted astrocytres) and oligodendrocytes (coiled bodies) prevalently located in basal ganglia and brainstem, while CBD is characterized by the presence of 4R tau in oligodendrocytes (coiled bodies), astrocytes (astrocytic plaques), and neurons located in neocortex and basal ganglia [3]. One of the most intriguing aspects of these diseases is therefore the fact that the same protein might account for different pathologies. Probably this is due to conformation-dependent changes in the protein affected. Indeed, either α-synuclein or tau can acquire different abnormal conformations, that can be considered analogous to the “strains” of prion diseases [3, 12–17]. It can be hypothesized that different strains of α-synuclein are responsible for PD or MSA while different strains of tau cause PSP or CBD. These structural differences might dictate specific tropism of these proteins for defined neuroanatomical regions or even cell types. Currently, both α-synuclein and tau are considered disease-specific biomarkers and definite diagnosis of PD and neurodegenerative parkinsonisms relies on their identification and anatomical distribution in brains collected at autopsy. Conversely, the in vivo diagnoses based on clinical criteria and neuroimaging are characterized by unsatisfactory sensitivity and specificity, and several cases might be misdiagnosed, especially in the early stages when clinical symptoms overlap [18–20]. Moreover, in some instances, the co-occurrence of different protein aggregates may take place in the brain. For instance, cases of PSP, CBD and AD confirmed at neuropathological level, were found to concurrently contain aggregates of α-synuclein in the form of Lewy bodies or, in other cases, aggregates of α-synuclein in isolation may phenotypically present as PSP or CBD [21–25]. Similarly, cases of MSA or PD were found to also contain aggregated species of tau [26–29]. The in vivo diagnostic accuracy for PD is at about 80 and 20% of patients classified as probable PD are indeed misdiagnosed [19]. Even worse, only 26% of patients with a diagnosis of possible PD are confirmed at autopsy [30]. The diagnostic accuracy of MSA is even lower and the clinical diagnosis is confirmed at autopsy in only 62% of patients [31–34]. Diagnostic accuracy of PSP has a specificity of about 95% for probable and 80–93% for possible cases [35–39] while only the 68% of patients diagnosed in vivo as CBD are confirmed at autopsy [40]. Therefore, a defined discrimination of these diseases is difficult with the current clinical diagnostic criteria alone. Compelling evidence suggests that trace amount of these abnormally folded proteins are present in cerebrospinal fluid (CSF) and peripheral tissues of diseased patients. Unfortunately, their concentration is well below the limits of detection of the conventional diagnostic techniques. By exploiting an innovative technique named Real Time Quaking Induced Conversion assay (RT-QuIC), developed in the field of prion diseases [41, 42], it was recently shown the presence of minute amount of abnormal α-synuclein in the CSF of PD patients with 95% sensitivity and 100% specificity [43]. Similarly, Soto and colleagues successfully identified α-synuclein in the CSF of patients with PD with 89% sensitivity and 97% specificity [44]. More recently, Groveman improved the assay and allowed quantitation of α-synuclein present in the CSF of patients with PD and Dementia with Lewy bodies (DLB) [45]. Finally, Saijo and colleagues showed the presence of abnormal 3R-tau in the CSF of patients with Frontotemporal dementia (FTLD) [46] with 100% sensitivity and 94% of specificity. Therefore, RT-QuIC represents a fundamental tool that might significantly increase the diagnostic accuracy of these diseases. The technique exploits the prion-like properties of abnormally folded α-synuclein and tau proteins, which become capable of interacting with their normal counterparts forcing them to acquire similar pathological structures. While in vivo this feature is considered to be responsible of the progression of α-synuclein or tau pathology within the brain (by transmission of protein misfolding), in vitro this property has been exploited in RT-QuIC for detecting minute amount of abnormally folded proteins in a known biological samples (CSF or blood or urine) [47–56]. In RT-QuIC, samples are incubated with a recombinant protein used as substrate of reaction. The presence of pathological proteins in the samples triggers the aggregation of the substrate, generating amyloid fibrils whose formation is monitored in real time with the use of the Thioflavin-T (ThT) fluorescent dye [57, 58]. Recent evidence has demonstrated that abnormal α-synuclein or tau proteins can accumulate in the olfactory epithelium collected post-mortem from patients with AD [59]. To the best of our knowledge, there are no reports that have evaluated the ability of RT-QuIC to detect trace amount of abnormally folded proteins in OM samples collected from patients with a clinical diagnosis of PD or neurodegenerative parkinsonisms. RT-QuIC has been successfully optimized for the analysis of OM samples collected from patients with prion diseases, so far [60]. We therefore decided to perform RT-QuIC experiments, using human recombinant α-synuclein (rec-αS) as reaction’s substrate, aimed at evaluating the presence of abnormal α-synuclein in OM samples collected from a consecutive series of patients affected by different neurodegenerative parkinsonian syndromes, to evaluate if seeding activity for α-synuclein can differentiate between synucleinopathies and tauopathies. Moreover, we evaluated if different strains of α-synuclein (MSA or PD) might have imprinted their aberrant structure to the same rec-αS used as RT-QuIC substrate. In contrast to the widely used CSF, OM samples can be periodically collected with a non-invasive procedure, thus representing optimal tissues for RT-QuIC analysis.

## Materials and methods

### Study design

This is a cross sectional observational study. A consecutive series of patients affected by neurodegenerative parkinsonisms were selected (Additional file 1: Table S1). Patients were eligible for enrollment if they had a diagnosis of idiopathic PD according to the Hughes and Postuma criteria [18, 61], MSA according to the Gilman criteria [33], PSP according to the Höglinger criteria [39] and CBD according to the Armstrong criteria [62]. Samples of olfactory mucosa were collected from each patients following well established procedure (referred to as “nasal brushing”) [63] which is shown at this link https://www.youtube.com/watch?v=wYb9W3u6uMY and described below. We selected only patients whose diagnosis was characterized by the highest clinical level of certainty.

### Expression and purification of human recombinant α-synuclein

Human recombinant α-synuclein (rec-αS) was cloned and expressed in pET-11a vector, using BL21 (DE3) *E. coli* strain. The expression of the protein was obtained by growing cells in Luria-Bertani broth medium with 100 mg/mL ampicillin at 37 °C, until an Optical density of about 0.6 at 600 nm, followed by induction with 0.6 mM isopropyl β-D-thiogalactoside (IPTG) for 5 h. The protein was subsequently extracted from bacterial periplasm by osmotic shock and boiling. Briefly, the cell pellet was incubated in osmotic shock buffer (30 mM Tris pH 7.2, 2 mM EDTA, 40% sucrose), followed by centrifugation (9000 rpm, 30 min) and boiling for 10 min. After two steps of ammonium sulfate precipitation (35 and 55%) the protein was purified by anion exchange chromatography (HiTrap column, GE Healthcare). AKTA purification systems (GE Healthcare) were used for monitoring the protein absorbance during chromatography process. The presence of rec-αS was monitored during all the purification steps by gel electrophoresis (SDS-PAGE). The identity and the purity of the final product was confirmed by Western blotting and mass spectroscopy. Fractions containing α-synuclein were dialyzed into water, quantified by measuring absorbance at 280 nm, lyophilized (FreeZone 2.5 Freeze Dry System, Labconco) and stored at − 80 °C. Before use, rec-αS was dissolved in H_2_O (at the final concentration of 5 mg/mL) and used to prepare the reaction mix.

### In vitro generation of recombinant α-synuclein aggregates

Rec-αS was diluted in a reaction mix composed of 40 mM PBS (pH 8.0), 170 mM NaCl and 10 μM Thioflavin-T (ThT) at the final concentration of 140 μM. Reactions were performed in triplicate in a black 96-well optical flat bottom plate (Thermo Scientific). Each well was supplemented with 100 μL of reaction mix. The plate was sealed with a sealing film (Thermo Scientific), inserted into a Fluoroskan Ascent microplate reader (Thermo Scientific) and subjected to cycles of shaking (1 min at 600 rpm, single orbital) and incubation (14 min at 42 °C). The addition of a 3-mm glass bead (Sigma) was required to sustain protein aggregation. The presence of protein aggregates was confirmed by means of ThT analysis, Western blot and Transmission Electron Microscopy (TEM) analyses.

### Preparation of brain samples for biochemical and RT-QuIC analyses

Frontal cortices of patients with neuropathologically confirmed diagnoses of Frontotemporal Dementia with parkinsonism-17 associated with P301L tau mutation (FTDP-17, *n* = 1), Progressive Supranuclear Palsy (PSP, *n* = 1), Corticobasal Degeneration (CBD, *n* = 1) and Non Demented Patient (NDP, *n* = 1); pons of patient with neuropathologically confirmed diagnosis of Parkinson’s disease (PD, *n* = 1); cerebellum of patient with neuropathologically confirmed diagnosis of Multiple System Atrophy (MSA, *n* = 1) were homogenized in PBS (pH 7.4, Sigma) at 10% (weight/volume), using a glass potter homogenizer. Samples were centrifuged (Eppendorf Centrifuge) at 800 × g, for 1 min at 4 °C, in order to remove cellular debris. Supernatants were collected and stored at − 20 °C for further biochemical and RT-QuIC analyses. For RT-QuIC analysis, serial dilutions of PD, MSA and FTDP-17 brain homogenates [10^-3^, 10^-6^ and 10^-9^] were prepared in PBS.

### Extraction of soluble and insoluble α-synuclein from brains

Extraction of soluble and insoluble α-synuclein fractions from brains was performed as previously described [64]. Briefly, approximately 0.3 g of frozen brain tissues were homogenized 10% (weight/volume) in TBS buffer (supplemented with protease inhibitors and phosphatase inhibitors), using a Tissue Lyser (Qiagen) with a steel bead, for 1 min at maximum speed. The homogenate was then clarified by centrifugation at 1000 x g for 5 min at 4 °C. Supernatants were transferred to polycarbonate centrifuge tubes and centrifuged at 100000 x g (Beckman Coulter Optima MAX) for 1 h at 4 °C. Resulting supernatants were collected as TBS-soluble fraction (soluble α-synuclein) while pellets were washed with 5 volumes of TBS and centrifuged at 100000 x g for 15 min at 4 °C. Pellets were then suspended in TBS buffer supplemented with SDS (5% final dilution) and sonicated (8 min at 500 W). Final solution was centrifuged at 100000 x g for 30 min at 25 °C. Supernatant was collected as SDS soluble fraction (detergent-soluble α-synuclein) while pellets were washed with 5 volumes of TBS-SDS buffer and centrifuged at 100000 x g for 15 min at 25 °C. Pellets were then suspended in 50 μL of TBS-SDS buffer supplemented with urea (8 M final concentration), sonicated for 5 min at 500 W, diluted 1:1 with TBS buffer and collected as urea soluble fraction (insoluble α-synuclein).

### SDS-PAGE and Western blotting

Brain extracts containing either soluble or insoluble α-synuclein were supplemented with LDS loading buffer, heated at 100 °C for 10 min and loaded into 12% Bolt Bis-Tris Plus gels (Invitrogen). Proteins were separated by means of SDS-PAGE and then transferred onto Polyvinylidene difluoride (PVDF) membranes (Immobilon-P, Millipore) and incubated with 5% (weight/volume) non-fat dry milk (prepared in Tris-HCl with 0.05% Tween-20) for 1 h at room temperature under shaking. PVDF membranes were incubated with primary antibody to α-synuclein (polyclonal AS08 358 Agrisera: epitopes 1–15) overnight at 4 °C under shaking.

RT-QuIC products were digested with proteinase K (see section PK digestions) and analyzed by Western blot following the same procedures described above. In this case, PVDF membranes were incubated with primary antibodies directed against different epitopes of α-synuclein (monoclonal 4D6 Invitrogen: epitopes 124–134 [65]; polyclonal AB5038 EMD Millipore: epitopes 111–131 [66]; monoclonal 5C2 Novus Biologicals: epitopes 61–95 [67]; polyclonal AS08 358 Agrisera: epitopes 1–15 [68]) overnight at 4 °C under shaking. Membranes were incubated with appropriate secondary antibodies conjugated with horseradish peroxidase (GE) and developed with chemiluminescent system (ECL Prime). Reactions were visualized using a G:BOX Chemi Syngene system.

### Collection of olfactory mucosa samples and preparation for RT-QuIC analyses

A total of 47 samples of olfactory mucosa (OM) were collected from: 18 patients with a clinical diagnosis of PD [18, 61], 11 patients with a clinical diagnosis of MSA [31], 6 patients with a clinical diagnosis of CBD [62] and 12 patients with a clinical diagnosis of PSP [37]. Before collection, the nasal cavity was treated with a topical anesthetic (Ecocain, Molteni Dental) for 10 min and, with the use of a special cotton swab (referred to as brush, FLOQSwabsTM Copan Italia, Brescia, Italy), OM were collected from the medial septal wall just above the middle turbinate, as previously described [63, 69]. After collection, cotton swabs were immersed in saline solution and olfactory cells separated from the brushes by means of vortexing. Cells were finally pelleted at 800 × g for 20 min at 4 °C. The supernatants were removed, and approximately 6 μg of the pellets were collected with the use of inoculating loops. Such material was then transferred into a tube containing 50 μL of PBS and used for RT-QuIC analyses.

### RT-QuIC analysis of in vitro generated α-synuclein aggregates

The solution containing in vitro generated α-synuclein aggregates was sonicated for 3 min at 500 W and serially diluted (from 10^− 1^ to 10^− 12^ volume/volume) in its own reaction buffer. Five μL of the following dilutions: undiluted, 10^− 3^, 10^− 6^, 10^− 9^, 10^− 12^ was added to 95 μL of reaction mix and subjected to RT-QuIC analysis. Reaction mix was composed as follow: rec-αS diluted in 40 mM PBS (pH 8.0), 170 mM NaCl and 10 μM Thioflavin-T (ThT) at the final concentration of 140 μM. All reagents used for the preparation of the reaction mix were filtered through a 0.22 μm filter before the addition of α-synuclein aggregates. All RT-QuIC reactions were performed in triplicate in a black 96-well optical flat bottom plate (Thermo Scientific) using the Fluoroskan Ascent microplate reader (Thermo Scientific). Samples underwent to cycles of shaking (1 min at 600 rpm, single orbital) and incubation (14 min at 42 °C). Fluorescent intensities, expressed as arbitrary units (AU), were taken every 30 min using 450 ± 10 nm (excitation) and 480 ± 10 nm (emission) wave-lengths, with a bottom read. The addition of a 3-mm glass bead (Sigma) was necessary to sustain protein aggregation.

### RT-QuIC analysis of brain homogenates

Brain homogenates (BH) collected from patients with PD, MSA, PSP, CBD, FTDP-17 and NDP were diluted at 10^− 3^ volume/volume in PBS. Samples were sonicated (3 min at 500 W) and 2 μL was then supplemented to 98 μL of RT-QuIC reaction mix prepared as follow: rec-αS diluted in 40 mM PBS (pH 8.0), 170 mM NaCl and 10 μM ThT at the final concentration of 70 μM. Reactions were performed in triplicate in a black 96-well optical flat bottom plate (Thermo Scientific). Each well contained 100 μL of final reaction’s volume. The plate was sealed with a sealing film (Thermo Scientific), inserted into a Fluoroskan Ascent microplate reader (Thermo Scientific) and subjected to cycles of shaking (1 min at 600 rpm, single orbital) and incubation (29 min at 42 °C). Fluorescent intensities, expressed as arbitrary units (AU), were taken every 30 min using 450 ± 10 nm (excitation) and 480 ± 10 nm (emission) wave-lengths, with a bottom read. The addition of a 3-mm glass bead (Sigma) was required to promote protein aggregation. A sample was considered “capable of seeding α-synuclein aggregation” if at least 2 out 3 replicates crossed a threshold of fluorescence set at 500 AU. We have then calculated the average fluorescence intensity of the two or three replicates that crossed this threshold and plotted resulting values in a graph against time. If only one (or none) of the replicates crossed the threshold, we considered the sample as “incapable of seeding α-synuclein aggregation” and we calculated the average fluorescence intensity of the two (or three) replicates that remained below such threshold.

### RT-QuIC analysis of olfactory mucosa samples

After preparation (as previously described), 2 μL of OM samples was added to 98 μL of RT-QuIC reaction mix which was prepared as follow: rec-αS diluted in 40 mM PBS (pH 8.0), 170 mM NaCl and 10 μM Thioflavin-T (ThT) at the final concentration of 140 μM. Reactions were performed in triplicate in a black 96-well optical flat bottom plate (Thermo Scientific). Each well contained 100 μL of final reaction’s volume. The plate was sealed with a sealing film (Thermo Scientific), inserted into a Fluoroskan Ascent microplate reader (Thermo Scientific) and subjected to cycles of shaking (1 min at 600 rpm, single orbital) and incubation (14 min at 42 °C). Fluorescent intensities, expressed as arbitrary units (AU), were taken every 30 min using 450 ± 10 nm (excitation) and 480 ± 10 nm (emission) wave-lengths, with a bottom read. The addition of a 3-mm glass bead (Sigma) was required to promote protein aggregation. A sample was considered “capable of seeding α-synuclein aggregation” if at least 2 out 3 replicates crossed a threshold of fluorescence set at 6 AU within a certain period of time set at 120 h. We have then calculated the average fluorescence intensity of the two or three replicates that crossed this threshold of fluorescence and plotted resulting values in a graph against time. If only one (or none) of the replicates crossed the threshold, we considered the sample as “incapable of seeding α-synuclein aggregation” and we calculated the average fluorescence intensity of the two (or three) replicates that remained below such threshold.

### PK digestion assays

Proteinase K (PK) was used to perform limited proteolytic digestion of the final RT-QuIC products (after seeding with brain homogenates or OM samples). In particular, samples were treated with PK 100 μg/mL at 37 °C for 60 min under shaking (500 rpm). Digestions were stopped by the addition of LDS loading buffer and boiling at 100 °C for 10 min. Western blot analyses were then performed.

### Transmission electron microscopy analyses

Ten μL of RT-QuIC products was dropped onto 200-mesh Formvar-carbon coated nickel grids for 30 min and the remaining drop was blotted dry using filter papers. The grids were subsequently stained with 25% Uranyl Acetate Replacement (UAR, negative staining) for 10 min, the solution was removed using filter papers and the grids were air-dried for 15 min before the analyses. Images were recorded at 120 kV with a FEI Tecnai Spirit, equipped with an Olimpus Megaview G2 camera.

### Statistical analyses

TEM images were analyzed with the Gwyddion software for measuring the distances between over-twists occurring in the same amyloid fibril. Final values were compared with a double-tailed unpaired t-test (Mann-Whitney U test) performed using the Prism software (GraphPad v5.0). Graphic representations of RT-QuIC kinetics (based on ThT signals) were also obtained with the Prism software (GraphPad v5.0). Densitometric analysis of PK resistant RT-QuIC products was performed using ImageJ software (v1.48) and final values were compared using a double tailed unpaired t-test (Mann-Whitney U test).

## Results

### In vitro generated α-synuclein aggregates efficiently seeded RT-QuIC reaction

**Fig. 1:**
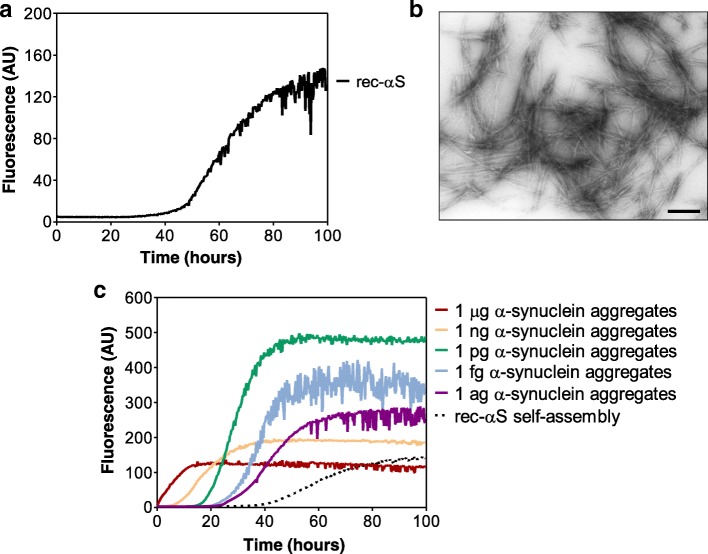
RT-QuIC analysis of in vitro generated α-synuclein aggregates. **a** In vitro generation of α-synuclein aggregates (*artificial seeds*). rec-αS [140 μM] was induced to aggregate by alternating cycles of incubation and shaking. Average ThT fluorescence intensity was plotted against time; **b** TEM analysis of final α-synuclein aggregates. Amyloid fibrils were efficiently generated in vitro under well controlled experimental conditions. Scale bar: 500 nm; **c** Assessment of the RT-QuIC detection limits. Serial dilutions of the *artificial seeds* previously produced were analyzed by means of RT-QuIC. All dilutions efficiently accelerated the kinetics of rec-αS aggregation. Average ThT fluorescence intensity was plotted against time. Self-assembly refers to unseeded rec-αS reactions

The kinetics of rec-αS aggregation was reproducible over time and with different batches of protein. Particularly, after a lag phase of about 40 h there was a rapid increasing in ThT signal that reached a plateau at around 100 h (Fig. 1a). TEM analysis of the products collected after 100 h confirmed the presence of α-synuclein aggregates, mostly in the form of amyloid fibrils (Fig. 1b). These aggregates were used as *artificial seeds*. In particular, they were serially diluted and added at the beginning of RT-QuIC assays performed using fresh rec-αS as reaction’s substrate. Results of these experiments are shown in Fig. 1c and clearly demonstrate that even the lowest dilution (which extrapolates to approximately 1 attogram of aggregated protein) was able to efficiently accelerate the kinetics of rec-αS aggregation mostly in a dose-dependent manner.

### Brain homogenates of patients with synucleinopathies efficiently seeded RT-QuIC reaction

**Fig. 2:**
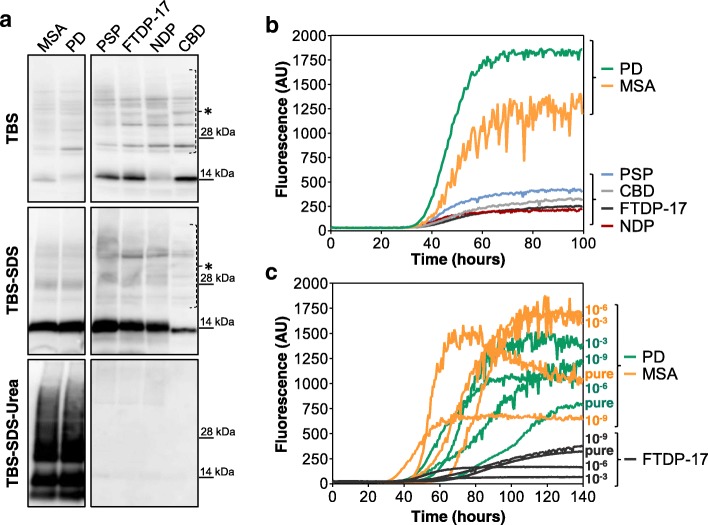
RT-QuIC analysis of brain homogenates of patients with PD and neurodegenerative parkinsonisms. **a** Extraction of soluble and insoluble α-synuclein fractions from brain homogenates of patients with PD, MSA, PSP, CBD, FTDP-17 or NDP control. Western blot analyses confirmed the presence of insoluble α-synuclein only in PD and MSA samples. Blots were immunostained with the AS08 358 antibody. Numbers in the right indicate the position of molecular weights. Asterisks indicate unspecific binding. **b** RT-QuIC analysis of BH samples. Two μL of sonicated BH collected from PD, MSA, PSP, CBD, FTDP-17 and NDP patients was added to rec-αS substrate and analyzed by means of RT-QuIC. PD and MSA samples efficiently induced rec-αS aggregation that reached higher levels of fluorescence intensities compared to those of PSP, CBD, FTDP-17 and NDP. Average ThT fluorescence intensity was plotted against time. **c** Assessment of the RT-QuIC detection limits. Serial dilutions (undiluted, 10^− 3^, 10^− 6^, 10^− 9^) of sonicated BH collected from PD, MSA and FTDP-17 subjects were analyzed by means of RT-QuIC. All dilutions efficiently induced rec-αS aggregation but those of FTDP-17 were characterized by lower fluorescence intensities compared to those of PD and MSA. Average ThT fluorescence intensity was plotted against time

Considering the high level of RT-QuIC sensitivity in detecting extremely low amounts of *artificial seeds*, we decided to verify its ability to detect pathological α-synuclein aggregates present in brain samples of patients with PD and MSA. Firstly, we have demonstrated the presence of α-synuclein aggregates in the brain of PD and MSA by means of Western blot. To this aim, brains of patients with MSA, PD, PSP, CBD, FTDP-17 and NDP were subjected to serial steps of high-speed centrifugation in order to separate soluble and insoluble (mostly aggregated) α-synuclein species. Subsequently, Western blot analyses confirmed the presence of aggregated forms of α-synuclein (urea-fraction) only in brain homogenates of patients with PD and MSA. No aggregates were detected in brain of patients with FTDP-17, PSP, CBD and NDP (Fig. 2a). Therefore, the same brain homogenates (10^− 3^ dilution) were analyzed by means of RT-QuIC. As shown in Fig. 2b, all brain samples were able to increase the kinetics of rec-αS aggregation with those of PD and MSA being characterized by higher fluorescence intensities (always above 1000 AU) compared to PSP, CBD, FTDP-17 and NDP (that never crossed 500 AU). Thus, considering a threshold of 500 AU we could identify brain homogenates of patients with PD and MSA. Since the amount of pathological α-synuclein eventually present in OM samples of PD and MSA patients is much lower than that present in the 10^− 3^ dilution of brain homogenates previously tested by means of RT-QuIC, we have performed additional experiments using lower dilutions [10^-6^ and 10^-9^] of PD and MSA samples. These dilutions were estimated to contain picograms [10^-3^], femtograms [10^-6^] and attograms [10^-9^] of pathological α-synuclein. As shown in Fig. 2c, all of them efficiently increased the kinetics of rec-αS aggregation. Notably, dilutions of FTDP-17 brain homogenates were used as controls and promoted rec-αS aggregation with less efficiency and lower fluorescence intensity (lower than 500 AU) than those of PD and MSA.

### Analysis of final RT-QuIC products seeded with different brain homogenates did not show biochemical differences

Being aware of the fact that different strains of α-synuclein (responsible for PD and MSA) [70] could force the same substrate to acquire distinct abnormal conformations, we decided to perform biochemical analysis of the final reaction products. In particular, RT-QuIC products seeded with PD, MSA and FTDP-17 brain homogenates were digested with PK and analyzed by means of Western blot. Regardless of the brain homogenate used to seed the reaction, all α-synuclein aggregates showed similar electrophoretic mobility and banding profile. Therefore, PK digestion alone was not able to demonstrate whether different seeds could imprint their specific conformations to rec-αS. Although characterized by low fluorescence intensity, also FTDP-17 brain homogenate revealed the presence of PK-resistant α-synuclein aggregates whose biochemical profile was similar to those of PD and MSA (Additional file 2: Figure S1a).

### Olfactory mucosa (OM) samples of patients with clinical diagnosis of PD or MSA efficiently seeded RT-QuIC reaction

**Fig. 3:**
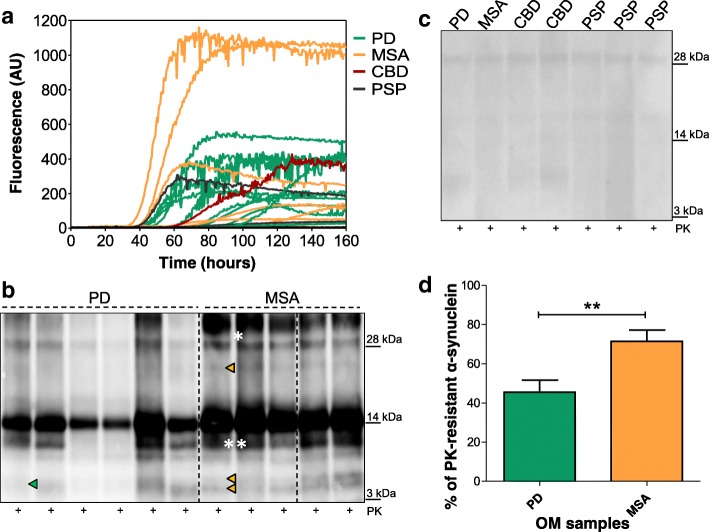
RT-QuIC analysis of OM samples collected from patients with PD and neurodegenerative parkinsonisms. **a** Kinetics of rec-αS aggregation after the addition of OM samples. Two μL of OM collected from PD (*n* = 18), MSA (*n* = 11), CBD (*n* = 6) and PSP (*n* = 12) was added to rec-αS substrate and analyzed by means of RT-QuIC. 10/18 samples of PD, 9/11 samples of MSA, 1/6 sample of CBD and 2/12 samples of PSP induced the aggregation of the substrate. Average ThT fluorescence intensity was plotted against time. **b** Biochemical analyses of RT-QuIC products of OM samples collected from PD and MSA patients that induced rec-αS aggregation (representative image). Ten μL of final RT-QuIC products were digested with PK and analyzed by means of Western blot. Green arrows indicate peculiar bands of RT-QuIC products seeded with PD samples. One band migrating at around 6–8 kDa is found in these samples. Orange arrows indicate peculiar band of RT-QuIC products seeded with MSA samples. Two bands are detected at around 6–8 kDa and a third band is detected at around 22 kDa. Blots were immunostained with the AS08 358 antibody. One asterisk (*) indicates the presence of aggregated species of α-synuclein, while two asterisks (**) indicate partially digested protein. Numbers in the right indicate the position of molecular weights. Dashed lines indicate cropped images from separate gels. **c** Biochemical analyses of RT-QuIC products of OM samples collected from PD, MSA, CBD and PSP patients that did not induce rec-αS aggregation. Ten μL of final RT-QuIC products were digested with PK and analyzed by means of Western blot and revealed the lack of PK-resistant bands. Blots were immunostained with the AS08 358 antibody. Numbers in the right indicate the position of molecular weights. **d** Densitometric analysis of RT-QuIC products seeded with PD (*n* = 4) or MSA (*n* = 4) samples. Three replicates per sample were subjected to PK treatment (100 μg/mL, 37 °C, 60 min) and immunostained with the AS08 358 antibody before quantification. This analysis confirmed that differences in PK resistance between PD and MSA samples were statistically significant (*p* = 0.0061)

OM samples collected from patients with a clinical diagnosis of PD (*n* = 18), MSA (*n* = 11), CBD (*n* = 6) and PSP (*n* = 12) (see Additional file 1: Table S1 for details) were blindly analyzed by means of RT-QuIC to investigate their effects on the kinetics of rec-αS aggregation. According to the thresholds of time (120 h) and fluorescence intensity (6 AU) set as described in materials and methods, we have found that 10 out of 18 samples of PD and 9 out of 11 samples of MSA were able to efficiently accelerate rec-αS aggregation (Fig. 3a). Similarly, 1 out of 6 samples of patients with CBD and 2 out of 12 samples of patients with PSP were able to trigger rec-αS aggregation. Hence, we have observed α-synuclein seeding activity in 19 out of 29 samples belonging to patients with probable α-synuclein pathology but also in 3 out of 18 samples belonging to patients with probable tauopathies (see Table 1). This may be due to the fact that CBD or PSP can be caused by distinct abnormal conformers of tau (strains) able to cross-seed the aggregation of α-synuclein with different efficiency. Otherwise, such seeding activity might suggest that these diseases have been misdiagnosed or they might have been correctly diagnosed but characterized by an incidental Lewy body deposition.

**Table 1:** Clinical data and OM/RT-QuIC results of all patients included in the study

	PD	MSA	CBD	PSP
Clinical criteria (ref.)	[18, 61]	[33]	[62]	[39]
Number of patients	18	11	6	12
Age at time of evaluation (years)	64.2 ± 7.8	62.3 ± 9.2	63.3 ± 10.6	68.3 ± 7.0
Age at disease onset (years)	52.4 ± 6.1	56.5 ± 9.5	60.2 ± 10.9	64.3 ± 8.2
Disease duration (years)	10.1 ± 5.1	5.8 ± 3.4	3.2 ± 1.6	4.0 ± 3.6
Gender (F/M)	8/10	5/6	4/2	5/7
Frequency of symptoms (%)				
• Rigid akinetic parkinsonism	100	90.1	83.3	91.7
• Tremor	88.9	81.8	50	8.3
• Ataxia	0	90.1	50	91.7
• Apraxia	0	0	100	33.3
• Delusions	16.7	9.1	0	8.3
• Dementia	11.1	0	16.7	58.3
• Psychiatric disorders	33.3	45.5	33.3	50
• REM behavioural disorder	55.6	63.6	0	0
• Autonomic impairment	83.3	100	33.3	16.7
RT-QuIC seeding activity for α-synuclein (% in total patients)	10 (56%)	9 (82%)	1 (16%)	2 (16%)

Values of continuous variables are presented as mean ± standard deviation (SD)

Notably, we did not find any correlation between positive RT-QuIC results and other clinical evaluations (especially disease-duration and age at disease onset) (Additional file 1: Table S1).

### OM samples of PD or MSA patients induced the formation of α-synuclein aggregates characterized by different biochemical features

Aggregated products resulting from RT-QuIC reactions seeded with OM samples of PD or MSA were collected at the end of the reaction and subjected to PK digestion (100 μg/mL) for 60 min at 37 °C. Digested samples where then analyzed by means of Western blot with the use of antibodies directed against different epitopes (C-terminal, Non-Amyloid-β Component (NAC) core and N-terminal part) of the protein (Additional file 2: Figure S1c). This enabled us to demonstrate that PK treatment efficiently removed the C-terminal part of the protein, while leaving a more resistant core composed of the NAC region and the most N-terminal part of α-synuclein, spanning from residues 1 to 15. Western blot analyses with anti NAC antibody (5C2) did not provide informative data other than demonstrating the presence of a PK resistant core. In contrast, analyses with antibody against the most N-terminal part of the protein demonstrated the presence of important and reproducible differences between samples seeded with olfactory mucosa of PD or MSA patients. In general, we have noted a higher resistance to PK digestion of all RT-QuIC products seeded with MSA samples compared to those seeded with PD. Densitometric analysis performed in triplicate on 4 PD and 4 MSA seeded samples confirmed that these differences were statistically significant (*p* = 0.0061) (Fig. 3d). Additionally, samples seeded with PD were characterized by the presence of a PK-resistant band migrating between 6 and 8 kDa, while samples seeded with MSA showed three PK-resistant bands, two migrating between 6 and 8 kDa, and a third one migrating at around 22 kDa (Fig. 3b). OM that did not induce α-synuclein aggregation, comprising some cases of PD or MSA and almost all PSP and CBD samples, were completely digested by PK and did not show any resistant band (Fig. 3c). Some PSP and CBD samples induced α-synuclein aggregation and their biochemical profile is reported in (Additional file 2: Figure S1b). In this case, both PSP and the CBD samples showed a PK-resistant α-synuclein banding profile typical of those observed in MSA seeded reactions.

### OM samples of PD or MSA patients induced the formation of α-synuclein aggregates characterized by different structural features

**Fig. 4:**
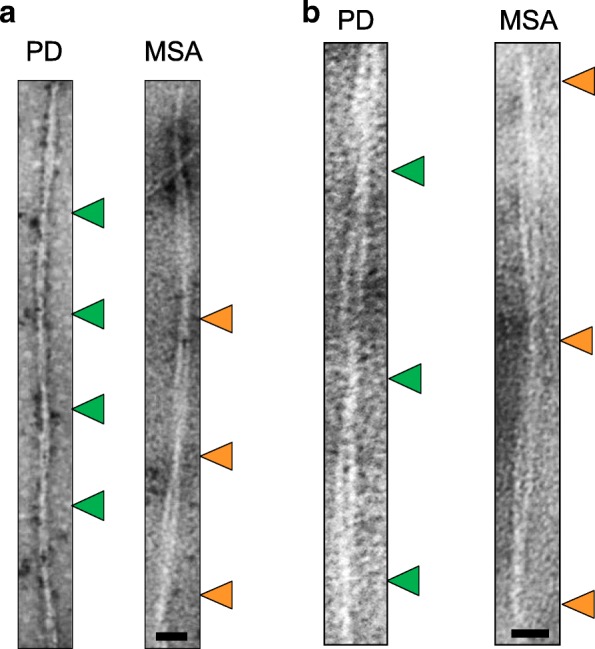
Representative TEM images of RT-QuIC products seeded with OM samples derived from PD and MSA patients. **a** Measurements of the distance between over-twists in final RT-QuIC fibrils seeded with samples of PD (*n* = 5) and samples of MSA (*n* = 5). As shown, the distance between over-twists in α-synuclein fibrils obtained from RT-QuIC products seeded with OM of MSA patients (orange arrows) was about 142 ± 1.3 nm (mean ± standard error of the mean) while that of PD patients (green arrows) was shorter and about 131 ± 1.1 nm and such differences were statistically significant (*p* < 0.0001, Mann-Withney U test). Scale bar: 35 nm. **b** TEM images of the same samples taken at higher magnification. Scale bar: 23 nm

Biochemical data were integrated with TEM analysis with the aim of verifying whether the differences in PK-resistant fragments were paralleled by morphological differences. Thus, we have analyzed the structural features of aggregated α-synuclein obtained at the end of the RT-QuIC assays seeded either with OM samples of PD (*n* = 5) or MSA (*n* = 5) patients. Shape, number and the length of fibrils were analyzed, with special focus on the presence and the distance between consecutive over-twists present in the same fibril. In total, we have analyzed the distance of over-twists present in 50 fibrils per patient, reaching a total number of 250 fibrils per pathology. These data were pooled together for each pathology (PD or MSA) and showed that the same α-synuclein substrate was surprisingly able to acquire different structural features when seeded with two distinct strains of α-synuclein. In particular, we have observed that the distance between over-twists in α-synuclein fibrils obtained from RT-QuIC products seeded with OM of MSA patients was about 142 ± 1.3 nm (mean ± standard error of the mean) while that of PD patients was shorter and about 131 ± 1.1 nm (Fig. 4a and b) and such differences were statistically significant (*p* < 0.0001, Mann-Withney U test). More interestingly, such differences were reproducible over time and within pathologies.

We have finally analyzed the RT-QuIC aggregates induced by some PSP or CBD samples and observed that the distance between over-twists was different from those of MSA or PD samples. In particular, those of PSP patients were at about 155 ± 1.5 nm, while those of CBD were at about 115 ± 1.7 nm (Additional file 3: Figure S2).

## Discussion

Definite diagnosis of PD or other neurodegenerative parkinsonisms is extremely challenging especially in the early stages of the disease when symptoms might overlap. These diseases are characterized by intracerebral accumulation of disease-specific abnormally folded proteins: α-synuclein in the case of PD and MSA, tau in the case of PSP and CBD. This process begins decades before the appearance of clinical signs of the disease. It is therefore conceivable that the abnormal proteins accumulate also in peripheral tissues and body fluids (e.g. olfactory mucosa, blood and urine) long before disease onset at concentrations well below the detection limits of the classical biochemical diagnostic techniques. In the last few years, an innovative technique, named RT-QuIC, has been developed in the field of prion diseases and enabled the detection of trace amounts of prions in CSF and peripheral tissues, in particular olfactory mucosa and skin samples with high levels of sensitivity and specificity [71]. Therefore, RT-QuIC has been proposed as diagnostic tool for prion diseases. Recently, it has been extended to the analysis of CSF samples collected from patients with other neurodegenerative disorders, including PD, DLB, AD and FTLD [43–46, 72]. Although with lower sensitivity and specificity compared to those reached in the field of prion diseases, RT-QuIC has successfully shown the presence of abnormal forms of α-synuclein, tau and Aβ in the CSF of PD or DLB, FTLD and AD patients.

Starting from these observations, we decided to optimize the RT-QuIC for the analysis of OM samples collected from patients with a clinical diagnosis of PD and MSA. As comparison, we have included OM samples from patients with clinical diagnosis of neurodegenerative parkinsonisms associated with tau pathology, i.e. PSP and CBD. Although data are currently available about RT-QuIC analysis for detecting abnormal α-synuclein in CSF [43–45] the study of OM samples might widen the diagnostic approach to these diseases by the analysis of tissues that can be collected by less invasive procedures.

The RT-QuIC analyses performed on α-synuclein are still in their embryonic phases and require further steps of standardization, reproducibility controls and harmonization before even thinking of introducing them as a diagnostic procedure. The main objective of our study was to verify whether OM collected from patients with PD and MSA have a different behavior as seeding activity for α-synuclein compared to patients with tauopathies.

First of all, we have set up the protocol of rec-αS aggregation and verified the ability of α-synuclein aggregates either artificially produced (referred to as *artificial seeds*) or present in brain samples to accelerate this kinetics. Our results showed an acceleration of rec-αS aggregation after the addition of attograms of *artificial seeds*, thus indicating that RT-QuIC is highly sensitive. The kinetics was also accelerated by the addition of PD, MSA, PSP, CBD, FTDP-17 and NDP brain homogenates, however the fluorescence intensities were significantly higher in reactions seeded with PD or MSA compared to the others. These data suggest that the homologous seeding (ability of abnormally folded α-synuclein to accelerate the aggregation of rec-αS) is more effective than the heterologous one where the kinetics of rec-αS aggregation could have been modified by proteins other than α-synuclein. This phenomenon is also known as *cross-seeding effect* and, for instance, abnormal forms of tau present in PSP, CBD and FTDP-17 samples could have contributed in stimulating rec-αS aggregation. Similarly, other proteins present in NDP sample might have sustained a cross-seeding effect. Since RT-QuIC analyses of brain homogenates enabled us to identify PD and MSA samples, we decided to verify whether this discrimination could also occur in RT-QuIC reactions seeded with OM collected from patients with clinical diagnosis of PD, MSA, PSP and CBD.

Compared to brains, where a faint cross-seeding effect often occurred, OM analysis produced much clear results. Probably OM samples contained fewer proteins or factors able to cross-seed rec-αS. PD and MSA samples were characterized by higher rec-αS seeding efficiency compared to PSP and CBD. In particular, almost all MSA samples (9/11) and more than half of PD samples (10/18) were able to induce rec-αS aggregation. Probably, the presence of abnormally folded α-synuclein in these samples was more efficient in stimulating rec-αS aggregation (homologous seeding). At difference, CBD and PSP samples did not induce aggregation of rec-αS, except for 2 out of 12 PSP and 1 out of 6 CBD. These results might have several explanations and here we report some of our hypotheses. First of all, since these pathologies are clinically diagnosed using criteria whose accuracy is not absolute, it might be that the clinical diagnosis was not correct. Other options to be considered include the fact that, as previously described, some of these diseases might share an incidental Lewy body deposition or that such phenomenon may be neither incidental nor coincidental, thus α-synuclein represents the unique pathological protein of otherwise usually tau-related clinical phenotypes [24]. Alternatively, some strains of tau might be more effective than others in cross-seed rec-αS aggregation, and the efficiency of such phenomenon may be amount-dependent [73–76]. Furthermore, OM is continuously regenerating and the amount of abnormally folded tau could depend on this process [77]. Unfortunately, collection and use of OM samples in the diagnostic field of neurodegenerative diseases is being born in the most recent years and it is not possible to perform retrospective analysis or compare data with neuropathological results to verify the sensitivity of the technique. For this reason, considered the limited number of samples analyzed, we decided not to calculate both sensitivity and specificity of the assay. Indeed, in contrast to prion diseases which have much shorter duration compared to PD and MSA, the autopsy confirmation might take many years. Therefore, we are aimed at collecting data and following up our patients for gathering information to be compared with neuropathological results in the future.

Notably, considering the accuracy of the in vivo diagnosis, a percentage of the patients of our case series might have been clinically misdiagnosed. Furthermore, regeneration of OM might influence the total amount of pathological α-synuclein present in the samples, thus decreasing RT-QuIC sensitivity. Recent evidence demonstrated an important drainage of CSF through the olfactory mucosa [78]. For this reason, our RT-QuIC results might have been influenced by a combination of both OM and CSF and their relative content of abnormally folded proteins. Another important point is that PD is characterized by a remarkable phenotypic heterogeneity that might be associated with different abnormal conformers of α-synuclein [15, 79–81]. Such strains might possess different seeding properties for rec-αS, thus explaining why some PD samples were not detected in our assay, in relation to the low sample size of the subjects evaluated. Moreover, the concomitant presence of other misfolded proteins (e.g. Aβ or tau) in OM samples might have influenced the aggregation properties of rec-αS.

Recent data from the literature have demonstrated that RT-QuIC might efficiently discriminate between Parkinson’s disease and other parkinsonisms [82, 83]. In some cases, the same RT-QuIC substrate can acquire distinct abnormal structures if supplemented with different seeds. Hence, we decided to verify whether OM of PD and MSA patients were able to induce the formation of α-synuclein aggregates characterized by disease-related biochemical and morphological features. Results of these analyses demonstrated that rec-αS acquired peculiar features when seeded with PD or MSA samples. In particular, α-synuclein fibrils produced by MSA showed three PK-resistant bands migrating at around 6–8, and 22 kDa and TEM analysis showed that these fibrils were characterized by the presence of over-twists whose distance was about 141 ± 1.3 nm (mean ± standard error of the mean). In contrast, α-synuclein fibrils produced by PD samples were significantly less resistant to PK digestion (*p* = 0.0061, Mann-Withney U test) and possessed one faint band migrating at around 6–8 kDa with distances between over-twists of about 131 nm ± 1.1 nm. These structural differences were statistically significant (*p* < 0.0001, Mann-Withney U test) and contribute in demonstrating that PD and MSA are caused by different strains of α-synuclein [70] that could effectively transmit their specific conformations to the same substrate. Moreover, differences in post-translational modifications of α-synuclein (e.g. phosphorylation) or in the size of the oligomeric α-synuclein seeds in PD and MSA might have influenced the RT-QuIC kinetics and the abnormal structures acquired by the substrate. Previous RT-QuIC experiments successfully demonstrated the presence of pathological α-synuclein in CSF samples collected from patients with PD and DLB, but none of them reported the possibility to recognize disease-specific abnormal protein conformers [43–46, 72].

The fact that our samples were collected from patients without neuropathological confirmation represents a relevant but at present not addressable limitation of our study. Vascular leukoencephalopathy was observed in 4 patients, who all received a clinical diagnosis of Parkinson’s disease according to Postuma criteria [18]. However, these patients, having evidence of only mild vascular disease and in cerebral regions unlikely associated with motor symptoms, did not meet criteria for vascular parkinsonism [84, 85] and were not excluded from the analysis. It is worth noting, however, that removal of these patients from analysis resulted in detection of α-synuclein seeding activity in 10 out of 14 OM samples, thus reaching an accuracy which is comparable to that of the RT-QuIC analysis of CSF [43, 44]. Such observations might be better defined in the future by using OM samples collected from patients neuropathologically verified.

## Conclusions

Our study provides the proof-of-concept that olfactory mucosa samples collected from patients with PD and MSA possess seeding activities for α-synuclein. These results represent a starting point for future studies aimed at [1] estimating sensitivity and specificity of RT-QuIC analysis on OM samples useful for PD and MSA diagnosis, [2] comparing the sensitivity of OM analysis with that of CSF simultaneously collected from the same patient, [3] integrating RT-QuIC analysis of CSF and OM with other instrumental and biochemical data and verifying whether this can significantly improve the clinical diagnostic accuracy of PD and other neurodegenerative parkinsonisms. Thus, if these observations will be confirmed and extended, α-synuclein RT-QuIC integrated with biochemical and TEM analyses may turn out to be a biomarker for the preclinical diagnosis of MSA and PD. Finally, RT-QuIC might offer a great opportunity to test the efficiency of several compounds to interfere with the process of rec-αS aggregation triggered by distinct α-synuclein strains, thus laying the foundations for a precision medicine.

## Additional files

Additional file 1: **Table S1.** Detailed clinical information of each patient and related RT-QuIC results of OM analyses. (XLSX 16 kb)

Additional file 2: **Figure S1.** Biochemical analyses of RT-QuIC products. **a** Western blot analyses of RT-QuIC aggregates seeded with 10^− 3^ dilutions of BH of PD, MSA and FTDP-17 subjects. Samples show the same banding profile. Blots were immunostained with the AS08 358 antibody. Numbers in the right indicate the position of molecular weights. **b** Western blot analyses of RT-QuIC aggregates seeded with OM samples of patients with tauopathies. Samples show a banding profile comparable to that of MSA seeded RT-QuIC reactions. Blots were immunostained with the AS08 358 antibody. Numbers in the right indicate the position of molecular weights. **c** Epitope mapping of RT-QuIC aggregates seeded with OM samples of patients with PD and MSA. C-terminal (4D6 and AB5038) antibodies did not detect any typical PK-resistant α-synuclein band associated with PD or MSA, while the NAC antibody (5C2) detected a faint PK resistant α-synuclein, especially in MSA seeded samples. Numbers in the right indicate the position of molecular weights. Dashed lines in **a**, **b** and **c** indicate cropped images from separate gels. (PDF 93 kb)

Additional file 3: **Figure S2.** (.pdf) TEM images of RT-QuIC products seeded with OM samples derived from CBD and PSP patients. Measurements of the distance between over-twists in final RT-QuIC products seeded with CBD (*n* = 1) and PSP (*n* = 1) samples and comparison with those obtained from PD and MSA patients. As shown, the distance between over-twists in α-synuclein fibrils obtained from RT-QuIC products seeded with OM of CBD (black arrows) and PSP (brown arrows) patients was about 115 ± 1.7 nm (mean ± standard error of the mean) and 155 ± 1.5 nm, respectively. Scale bar: 35 nm. (PDF 30 kb)

## Data Availability

All data generated or analyzed during this study are included in this published article and its supplementary information files.

## Abbreviations

4R: 4-repeat tau
AD: Alzheimer’s disease
AU: Arbitrary unit
BH: Brain homogenate
CBD: Corticobasal degeneration
CNS: Central nervous system
CSF: Cerebrospinal fluid
DLB: Dementia with Lewy bodies
FTDP-17: Frontotemporal dementia with parkinsonism-17 associated with P301L tau mutation
FTLD: Frontotemporal lobar degeneration
MSA: Multiple system atrophy
NAC: Non-amyloid-β component
NDP: Non demented patient
OM: Olfactory mucosa
PD: Parkinson’s disease
PK: Proteinase K
PSP: Progressive supranuclear palsy
PVDF: Polyvinylidene difluoride
rec-αS: recombinant α-synuclein
RT-QuIC: Real Time Quaking Induced Conversion
SDS-PAGE: SDS-polyacrylamide gel electrophoresis
TEM: Transmission electron microscopy
ThT: Thioflavin T
