# Supplementary figures and images for: Efficient RT-QuIC seeding activity for α-synuclein in olfactory mucosa samples of patients with Parkinson’s disease and multiple system atrophy
Source: Transl Neurodegener. 2019 Aug 8;8:24. doi: 10.1186/s40035-019-0164-x (PMC6686411; doi:10.1186/s40035-019-0164-x)

## Additional file 2: Figure S1

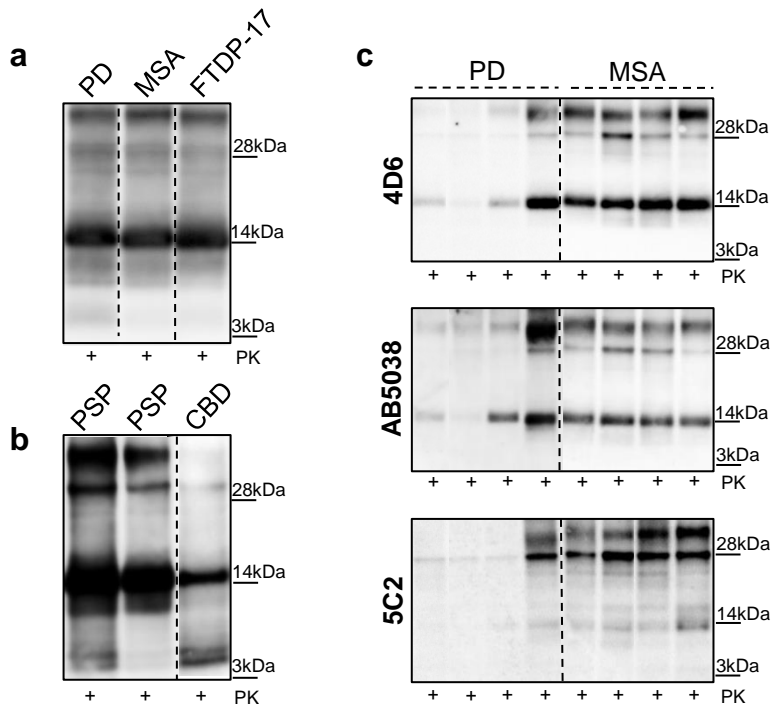

Supplement: Supplementary file 2 — Figure S1. Biochemical analyses of RT-QuIC products. a Western blot analyses of RT-QuIC aggregates seeded with 10− 3 dilutions of BH of PD, MSA and FTDP-17 subjects. Samples show the same banding profile. Blots were immunostained with the AS08 358 antibody. Numbers in the right indicate the position of molecular weights. b Western blot analyses of RT-QuIC aggregates seeded with OM samples of patients with tauopathies. Samples show a banding profile comparable to that of MSA seeded RT-QuIC reactions. Blots were immunostained with the AS08 358 antibody. Numbers in the right indicate the position of molecular weights. c Epitope mapping of RT-QuIC aggregates seeded with OM samples of patients with PD and MSA. C-terminal (4D6 and AB5038) antibodies did not detect any typical PK-resistant α-synuclein band associated with PD or MSA, while the NAC antibody (5C2) detected a faint PK resistant α-synuclein, especially in MSA seeded samples. Numbers in the right indicate the position of molecular weights. Dashed lines in a, b and c indicate cropped images from separate gels. (PDF 93 kb) [file 40035_2019_164_MOESM2_ESM.pdf]

Additional file 3: Figure S2

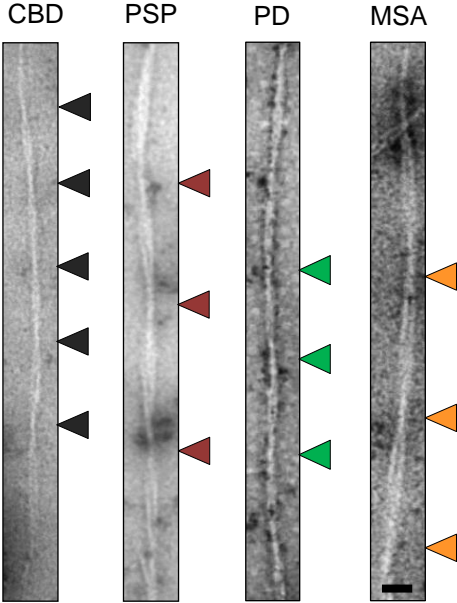

Supplement: Supplementary file 3 — Figure S2. (.pdf) TEM images of RT-QuIC products seeded with OM samples derived from CBD and PSP patients. Measurements of the distance between over-twists in final RT-QuIC products seeded with CBD (n = 1) and PSP (n = 1) samples and comparison with those obtained from PD and MSA patients. As shown, the distance between over-twists in α-synuclein fibrils obtained from RT-QuIC products seeded with OM of CBD (black arrows) and PSP (brown arrows) patients was about 115 ± 1.7 nm (mean ± standard error of the mean) and 155 ± 1.5 nm, respectively. Scale bar: 35 nm. (PDF 30 kb) [file 40035_2019_164_MOESM3_ESM.pdf]
